# SARS‐CoV‐2 PL^pro^ Inhibition: Evaluating in Silico Repurposed Fidaxomicin's Antiviral Activity Through In Vitro Assessment

**DOI:** 10.1002/open.202400091

**Published:** 2024-08-05

**Authors:** Sara Protić, Milica Crnoglavac Popović, Nevena Kaličanin, Olivera Prodanović, Milan Senćanski, Jelena Milićević, Kristina Stevanović, Vladimir Perović, Slobodan Paessler, Radivoje Prodanović, Sanja Glišić

**Affiliations:** ^1^ Faculty of Chemistry University of Belgrade Studentski Trg 12–16 Belgrade Serbia; ^2^ Institute of Chemistry Technology and Metallurgy University of Belgrade Njegoševa 12 Belgrade Serbia; ^3^ Institute for Multidisciplinary Research University of Belgrade Kneza Višeslava 1 Belgrade Serbia; ^4^ Laboratory of Bioinformatics and Computational Chemistry Institute of Nuclear Sciences Vinca National Institute of the Republic of Serbia University of Belgrade Mike Petrovica Alasa 12–14 Belgrade Serbia; ^5^ Department of Pathology University of Texas Medical Branch Galveston Texas United States; ^6^ Institute for Human Infections and Immunity University of Texas Medical Branch Galveston Texas United States; ^7^ Laboratory for Plant Molecular Biology Institute of Molecular Genetics and Genetic Engineering University of Belgrade Vojvode Stepe 444a Belgrade Serbia

**Keywords:** antiviral therapy, drug repurposing, fidaxomicin, SARS-CoV-2 PLpro, *in vitro*

## Abstract

The emergence of drug‐resistant viruses and novel strains necessitates the rapid development of novel antiviral therapies. This need was particularly demanding during the COVID‐19 pandemic. While de novo drug development is a time‐consuming process, repurposing existing approved medications offers a more expedient approach. In our prior in silico screening of the DrugBank database, fidaxomicin emerged as a potential SARS‐CoV‐2 papain‐like protease inhibitor. This study extends those findings by investigating fidaxomicin‘s antiviral properties in vitro. Our results support further exploration of fidaxomicin as a therapeutic candidate against SARS‐CoV‐2, given its promising in vitro antiviral activity and favorable safety profile.

## Graphical abstract

Drug repurposing is the most suitable approach in emergency cases, like novel virus pandemics. Our well setup machinery, including combined computational and wet lab approaches, managed to output a new antiviral candidate.

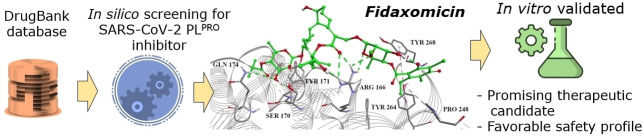

## Introduction

The emergence and rapid spread of COVID‐19 have led to a global effort to develop effective therapeutics. Significant progress has been made in this endeavor, with the authorization of several antiviral drugs, monoclonal antibodies, and immunomodulatory therapies.^[1]^ However, despite these advancements, several challenges persist. Future treatment strategies for COVID‐19 need to demonstrate efficacy and capability of addressing the threat of drug resistance arising from new viral variants.

The recent emergence of orally available antiviral medications, nirmatrelvir (Paxlovid)^[1]^ and molnupiravir, represents a significant breakthrough in managing COVID‐19. These drugs offer promising treatment options, especially for vulnerable populations like the immunocompromised, elderly, and children, who may not achieve adequate protection from vaccination.^[2]^ Nirmatrelvir, developed by Pfizer, targets a critical viral enzyme, the main protease (M^pro^). It has received emergency use authorization (EUA) from the FDA in combination with ritonavir.^[2]^ Similarly, Merck's molnupiravir targets the viral RNA replication machinery. While approved for emergency use as an oral therapy for adults, limitations exist.^[3]^ Paxlovid may interact with other medications, and molnupiravir‘s efficacy is lower than initially anticipated, necessitating careful monitoring.[^4^, ^5^] Additionally, emerging reports suggest resistance to nirmatrelvir against specific mutations in the M^pro^ site of the Omicron variant.^[6]^ These limitations highlight the need to develop new and improved COVID‐19 antiviral drugs with enhanced efficacy, reduced side effects, and broader applicability.

SARS‐CoV‐2, the causative agent of COVID‐19, possesses a single‐stranded, positive‐sense RNA genome encompassing at least ten open reading frames (ORFs).^[7]^ The primary ORF, known as ORF1ab, constitutes approximately two‐thirds of the viral genome and encodes two pivotal overlapping polyproteins, pp1a and pp1ab.^[7]^ These polyproteins play indispensable roles in viral replication and transcription, undergoing cleavage by virally encoded cysteine proteases to yield 16 non‐structural proteins (NSPs).[^7^, ^8^, ^9^] One of these proteases, papain‐like protease (PL^pro^), is encoded within NSP3. PL^pro^ exhibits specificity towards a distinct sequence motif (LXGG tetrapeptide) situated among various viral proteins, cleaving the replicase polyproteins at their N‐termini to release essential individual NSPs, including nsp1, nsp2, and nsp3, vital for viral replication.[^10^, ^11^] Moreover, PL^pro^ contributes to the evasion of the host‘s antiviral immune response by deubiquitinating and deISGylating host cell proteins.^[12]^ Consequently, the inhibition of PL^pro^ emerges as a promising therapeutic approach for COVID‐19 patients, given its pivotal role in viral replication and immune evasion.

The central challenge posed by the COVID‐19 pandemic underscores the pressing demand for effective preventive and therapeutic measures. Recognizing the considerable time required to develop entirely new drugs, repurposing existing medications presents an appealing approach to address therapeutic needs swiftly.

A recent study found that Fidaxomicin inhibits RNA synthesis by the SARS‐CoV‐2 RNA‐dependent RNA polymerase (RdRp) in vitro and suppresses viral replication in cell culture.^[13]^ Additionally, an *in silico* repurposing study identified Fidaxomicin as a candidate SARS‐CoV‐2 Papain‐like Protease (PL^pro^) candidate inhibitor.^[14]^ Expanding upon this promising dual targeting potential and considering the established benefits of combination therapies in antiviral strategies, we investigated fidaxomicin‘s ability to inhibit PL^pro^ in vitro. The findings of our study confirm fidaxomicin‘s activity against PL^pro^, supporting its further exploration as a potential therapeutic candidate for COVID‐19.

## Results and Discussion

This study aimed to validate fidaxomicin previously *in silico*‐identified inhibitory potential against PL^pro^.^[14]^ Previously, in our initial study findings, we reported docking energies that suggested favorable interactions with PL^pro^′s active site. Here, we present the docking results with a visualization, followed by detailed descriptions of our in vitro experiments to elucidate fidaxomicin‘s inhibitory mechanism and potency against SARS‐CoV‐2 PL^pro^ activity. This sequential approach ensures clarity and strengthens our experimental design by incorporating structural insights and background information.

### Molecular Docking

**Figure 1:**
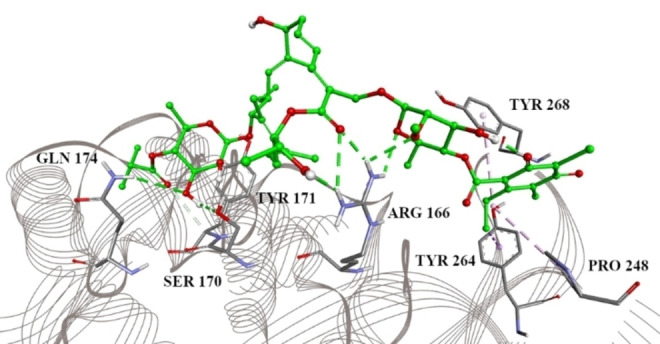
Fidaxomicin in the PL^pro^ inhibitor binding site, with marked interacting amino acid residue. Green lines: hydrogen bonds; light green: carbon hydrogen bond; purple: alkyl‐π/hydrophobic interactions; magenta: π‐σ interactions.

Using the PL^pro^‐GRL 0617 complex structure (PDB ID 7CJM),^[15]^ we performed molecular docking into the binding site of reported co‐crystallized PL^pro^ inhibitors in our earlier study.^[14]^ The binding site was determined according to the intermolecular interactions with co‐crystalized ligand GRL 0617^[15]^ and additionally confirmed using our ISM approach (Supplementary Figure S1).^[14]^ Fidaxomicin forms important (alkyl‐π/hydrophobic or π‐σ) interactions with Tyr 268, Tyr 264, and Pro 248 as shown in Figure 1. On the other side of the binding site, however, it is stabilized by a network of hydrogen bonds with Arg 166, Ser 170, Tyr 171, and Gln 174. This finding revealed the potential of fidaxomicin‘s inhibitory action on PL^pro^.

### Purification of Papain‐Like Protease

**Figure 2:**
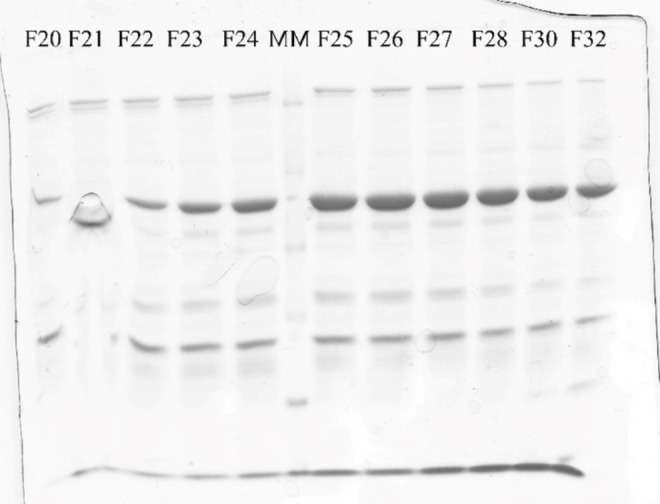
Polyacrylamide gel electrophoresis (SDS‐PAGE) on 10 % separating gel of purified papain‐like protease (F20–F24); (MM) molecular weight markers (14.4–116 kDa); fractions of purified papain‐like protease (F25–F32).

Papain‐like protease was expressed in *Escherichia coli* BL21 STAR cells and purified to homogeneity by the Ni‐NTA affinity chromatography. The process of purification was analyzed by SDS electrophoresis on a 10 % gel. The estimated molecular mass of the purified protein is approximately 60 kDa (Figure 2), which is in accordance with the literature data for the weight of one GST‐tagged papain‐like protease subunit, 63.9 kDa.

### Papain‐Like Protease Assay

**Figure 3:**
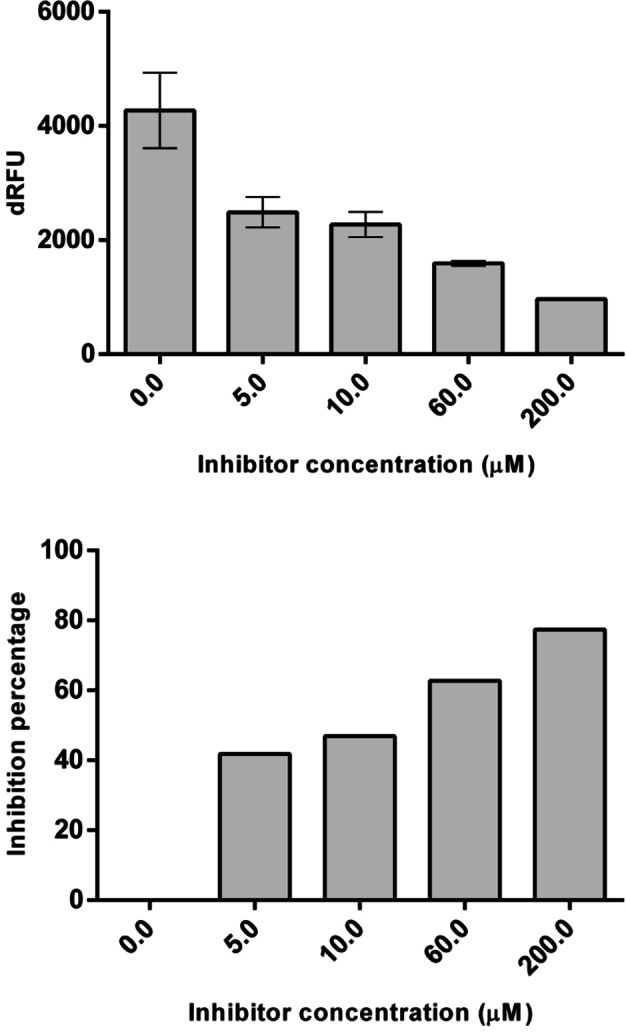
Relative activity (dRFU) and inhibition percentage (%) of PL^pro^ with different concentrations of fidaxomicin.

The results of the present study show that fidaxomicin has inhibitory activity against PL^pro^, and that 50 % of inhibition was achieved at around 10 μM concentration (Figure 3, Table 1).

**Table 1:** Kinetic data for the inhibition of PL^pro^ by fidaxomicin.

I (mM)	dRFU	stdev	Stdev (%)	Residual activity (%)	Inhibition (%)
0	4275	660.3	15.45	100	0
0.005	2488	268.0	10.77	58.2	41.8
0.01	2272	219.2	9.648	53.2	46.8
0.06	1594	39.60	2.484	37.3	62.7
0.2	966.5	3.535	0.3658	22.6	77.4

## Discussion

The need for novel antiviral drugs to combat SARS‐CoV‐2 infection was urgent and increasingly apparent. This urgency was driven by the emergence of resistant viral strains and the limitations of existing treatment strategies. Substantial attention has been given to drug repurposing, which offers a promising approach to addressing the challenges posed by COVID‐19. Leveraging *in silico* approaches enables the rapid screening of extensive libraries of existing compounds, providing a crucial advantage when time is of the essence. SARS‐CoV‐2 papain‐like protease (PL^pro^) is a critical enzyme for viral replication and, although no inhibitors are currently approved, it remains a focus for drug development efforts.^[16]^

In our previous study, we employed a combined virtual screening approach to identify potential inhibitors of SARS‐CoV‐2 PL^pro^. We have used the Informational spectrum method (ISM) for the structure/function analysis of the SARS‐CoV‐2 protein PL^pro^.[^17^, ^18^] Further utilizing the Informational Spectrum Method for Small Molecules (ISM‐SM), a method to identify ligand‐target interactions based on spectral fingerprints, we explored the Drugbank database and, through subsequent molecular docking, identified fidaxomicin as a candidate PL^pro^ inhibitor.^[14]^ The ISM‐SM presents a unique advantage compared to other computational approaches due to its capability to recognize extensive molecular interactions and connections between proteins and ligands across distances ranging from 5 to 1000 Å.^[19]^ This method accurately pinpoints the positions of protein domains likely involved in binding, facilitating the selection of small molecules tailored to these domains with high precision. Moreover, swiftly screening large compound libraries is straightforward, necessitating only the protein sequence and SMILES notation of molecules for data preparation. Recently, by employing the same combined virtual screening (VS) protocol utilized in this study, we identified potential SARS‐CoV‐2 Mpro inhibitors and suggested 57 compounds for subsequent experimental evaluation.^[20]^ Additionally, this approach has been used effectively in analyzing GPCR drugs from the Golden dataset.^[19]^ This study builds upon our prior findings, where ISM‐SM was employed in combination with ligand‐based virtual screening and molecular docking to propose fidaxomicin as a PL^pro^ inhibitor.^[14]^

At the onset of the pandemic, the use of ISM for the first time led to the identification of a potential SARS‐CoV‐2 receptor and proposed therapeutic/vaccine targets, as well as elucidating SARS‐CoV‐2 cell‐to‐cell transmission mechanisms.^[21]^

Here, our objective is to validate the potential antiviral activity of fidaxomicin in vitro. Our experiments demonstrate the measurable anti‐SARS‐CoV‐2 activity of fidaxomicin in cell culture.

The present study makes two pivotal contributions: first, it showcases the in vitro anti‐SARS‐CoV‐2 efficacy of the established drug fidaxomicin, and second, it validates prior *in silico* drug repurposing efforts, pinpointing fidaxomicin as a candidate PL^pro^ inhibitor.

Several *in silico* studies have highlighted fidaxomicin as a potential candidate for combating SARS‐CoV‐2, albeit targeting different viral components. Parvez et al.^[22]^ focused on the virus‘s RNA‐dependent RNA polymerase (RdRp) and identified fidaxomicin through computational methods as having high binding affinity, indicating its potential for repurposing as a COVID‐19 treatment. Similarly, Guedes et al.^[23]^ found fidaxomicin to have promising docking scores with the virus‘s N protein, although they stress the need for experimental validation to confirm its antiviral effect. Gangadharan et al.^[24]^ identified fidaxomicin as a potent inhibitor of SARS‐CoV‐2 RdRp based on computational analysis, further supporting its potential in combating the virus.

Fidaxomicin is an approved macrocyclic lactone antibiotic drug for the treatment of Clostridium difficile infection, indicating its safety and tolerability profile in humans. Fidaxomicin targets the bacterial RNA synthase, exhibiting activity against Clostridium difficile.^[25]^

Fidaxomicin, besides being a well‐established antibiotic, presents a promising candidate for repurposing as a broad‐spectrum antiviral agent. This potential is supported by its ability to inhibit the replication of multiple viruses, including Zika (ZIKV) and dengue virus, in both cell culture and animal models. Regarding its underlying mechanism, fidaxomicin appears to target the ZIKV NS5 protein, hindering its RNA synthesis. Furthermore, recent studies already mentioned have demonstrated that fidaxomicin can inhibit RNA synthesis by the SARS‐CoV‐2 virus, the causative agent of COVID‐19, in cell cultures, potentially suppressing viral replication.^[13]^ These findings and the ZIKV and dengue virus results collectively suggest a broad‐spectrum antiviral potential for fidaxomicin, warranting further investigation.^[26]^

Besides the previous findings of fidaxomicin‘s ability to inhibit viral RNA synthesis (RdRp),^[13]^ we identified fidaxomicin as a candidate inhibitor of the SARS‐CoV‐2 Papain‐like Protease (PL^pro^) through computational analysis.^[14]^ This article confirms the in vitro activity of fidaxomicin against PL^pro^, suggesting dual targeting Sarcov2 drug that can potentially mitigate the emergence of drug resistance, a significant concern in viral treatment. These findings warrant further *in vivo* assessment to explore fidaxomicin‘s therapeutic potential in combating COVID‐19. This could involve evaluating its antiviral activity in relevant animal model systems closely mimicking human infection. One possible direction for *in vivo* experiments is using the hamster model, which has shown promise in replicating the pathophysiology of COVID‐19 in humans.

## Conclusions

Exploring novel therapeutic avenues is crucial for achieving successful treatment outcomes in the face of the ever‐growing challenge of drug resistance. This study investigated fidaxomicin‘s in vitro antiviral potential against SARS‐CoV‐2. Our findings could contribute to the urgent need for effective COVID‐19 treatments with minimal side effects. This research may serve as a starting point for future exploration, providing insights that could assist healthcare systems in navigating emerging viral threats and addressing future public health challenges.

## Experimental Section

### Molecular Docking

Molecular docking of the candidates into the crystal structure of PL^pro^ was carried. Receptor three‐dimensional structure was downloaded from RCSB, PDB ID 7CJM.^[15]^ All ligands, waters and ions were removed from PDB file. The grid box with dimensions 24×24×24 Å was set to span all amino acid residues interacting with co‐crystallised inhibitor GRL 0617. The (x,y,z) center of the grid box was (26.0, 70.0, −1.0). Selected drug was converted from SMILES to 3D SDF and further to PDB files and protonated at physiological pH. Geometry optimization was carried in MOPAC 2016^[27]^ at PM7^[28]^ level of theory. Default software settings for hydrophobic and hydrophilic terms in docking search function were used. Exhaustiveness was set to 50. Molecular docking was carried in Autodock Vina 1.1.2.^[29]^

Figures were made in BIOVIA Discovery Studio 2017 and Origin 9.0 software.

### Equipment

The thermostat “Environmental Shaker‐Incubator ES‐20” and the shaker “Thermo shaker TS‐100 Biosan” were used for the needs of growing microorganisms. The “Consort E122” system was used for protein electrophoresis. HPLC AKTA system was used for enzyme purification. TEKAN Infinite 200 Pro M Nano+ device was used to measure the enzyme activity by fluorescence.

### Chemicals

Kanamycin, an antibiotic, was ordered from Invitrogen, California. Agar, peptone, and tryptone, components for media preparation, were ordered from Torlak, Serbia. Other substances were ordered from Centrohem, Serbia.

### Gene for Papain‐Like Protease

The gene for papain‐like protease was ordered from Addgene, and cloned into the pETM33 vector with N‐terminal GST and His‐tag.

Gene for papain‐like protease was cloned using NcoI and EcoRI restriction enzymes. These restriction sites were destroyed during cloning. Himera: His‐GST‐HRV_3C‐PLP has 1677 bp with a Mr of 63.9 kDa. The recommended expression conditions when using *E. coli* BL21 DE3 gold cells are growth at 37 °C, induction with IPTG in a final concentration of 1 mM, and expression for 16 h at 18 °C. *E. coli* STAR strain was used for intracellular expression. Zink‐acetate was added to the expression media at a final concentration of 0.5 mM. The DH5α strain was used for the storage and propagation of plasmids.

### Amino Acid Sequence of Papain‐Like Protease

DGEVRTIKVFTTVDNINLHTQVVDMSMTYGQQFGPTYLDGADVTKIKPHNSHEGKTFYVLPNDDTLRVEAFEYYHTTDPSFLGRYMSALNHTKKWKYPQVNGLTSIKWADNNCYLATALLTLQQIELKFNPPALQDAYYRARAGEAANFCALILAYCNKTVGELGDVRETMSYLFQHANLDSCKRVLNVVCKTCGQQQTTLKGVEAVMYMGTLSYEQFKKGVQIPCTCGKQATKYLVQQESPFVMMSAPPAQYELKHGTFTCASEYTGNYQCGHYKHITSKETLYCIDGALLTKSSEYKGPITDVFYKENSYTTT

### Isolation and Purification

#### Cell lysis

After the protein expression, the collected cells were resuspended in lysis buffer (50 mM Na‐phosphate buffer with 300 mM NaCl and 10 mM imidazole, pH 7.5). The sample was sonicated on ice with an ultrasound probe 10 times for 10 s, with a 20‐s pause in between. After lysis, the cells were centrifuged for 20 min at 13,000 rpm. The supernatant was passed through a sterile 0.22 μL filter.

#### Purification

PL^pro^ was purified by an HPLC system on a 5 mL Ni‐NTA FF Sepharose column. For column equilibration, a 50 mM Na‐phosphate buffer with 300 mM NaCl and 10 mM im‐idazole, pH 7.5, was used, while the same buffer with a gradient from 10 mM to 350 mM imidazole was used for protein elution. The change at 280 nm was monitored. Fractions of the purified protein were checked by SDS‐PAGE electrophoresis (Figure 2). Fractions containing the pure PL^pro^ (fractions 22–28) were pulled and dialyzed against a 50 mM Tris‐HCl buffer, pH 7.5, with 150 mM NaCl and 1 mM DTT (Figure 2). The isolated enzyme was stored in 10 % glycerol at −20 °C.

#### Papain‐Like Protease Assay

The change in fluorescence was monitored for 35 minutes every 5 min, with excitation at 485 nm and emission at 535 nm. The total volume of the reaction mixture was 200 μl. The buffer in which the reaction took place was 20 mM Tris with 150 mM NaCl and 1 mM DTT pH 7.5. The enzyme (10 μl) and 0.3 μl of the fluorescent substrate (Recombinant Human Ubiquitin Rhodamine 110 Protein) dissolved in DMSO were added to the reaction mixture so that the final substrate concentration was 0.375 μM. Fidaxomicin (5 μM, 10 μM, 60 μM and 200 μM), dissolved in DMSO, was used as a possible inhibitor.

## Conflict of Interests

The authors declare no conflict of interest.

1

## Supporting information

As a service to our authors and readers, this journal provides supporting information supplied by the authors. Such materials are peer reviewed and may be re‐organized for online delivery, but are not copy‐edited or typeset. Technical support issues arising from supporting information (other than missing files) should be addressed to the authors.

Supporting Information

## Data Availability

The data that support the findings of this study are available from the corresponding author upon reasonable request.

## References

[1] G. Li , R. Hilgenfeld , R. Whitley , E. De Clercq , Nat. Rev. Drug Discovery 2023, 22, 449. 37076602 10.1038/s41573-023-00672-y PMC10113999

[2] Y. N. Lamb , Drugs 2022, 82, 585. 35305258 10.1007/s40265-022-01692-5 PMC8933659

[3] T. K. Burki , Lancet Respir. Med. 2022, 10, e18.35033223

[4] M. Kozlov , Nature 2021, DOI 10.1038/d41586-021-03667-0.

[5] J. Heskin , S. J. C. Pallett , N. Mughal , G. W. Davies , L. S. P. Moore , M. Rayment , R. Jones , The Lancet 2022, 399, 21. 10.1016/S0140-6736(21)02657-X PMC8718360 34973713

[6] S. Chatterjee , M. Bhattacharya , K. Dhama , S.-S. Lee , C. Chakraborty , Mol. Ther. Nucleic Acids 2023, 32, 263. 37041859 10.1016/j.omtn.2023.03.013 PMC10078092

[7] Y. A. Malik , Malays. J. Pathol. 2020, 42, 3. 32342926

[8] L. Mousavizadeh , S. Ghasemi , J. Microbiol. Immunol. Infect. 2021, 54, 159. 32265180 10.1016/j.jmii.2020.03.022 PMC7138183

[9] Y. Qiu , K. Xu , STEMedicine 2020, 1, e39.

[10] C. B. McClain , N. Vabret , Signal Transduct. Target. Ther. 2020, 5, 1. 33024071 10.1038/s41392-020-00335-z PMC7537779

[11] G. Sun , L. Xue , Q. He , Y. Zhao , W. Xu , Z. Wang , Stem Cell Res. 2021, 52, 102219. 33550140 10.1016/j.scr.2021.102219 PMC7985237

[12] S. G. Devaraj , N. Wang , Z. Chen , Z. Chen , M. Tseng , N. Barretto , R. Lin , C. J. Peters , C.-T. K. Tseng , S. C. Baker , K. Li , J. Biol. Chem. 2007, 282, 32208. 17761676 10.1074/jbc.M704870200 PMC2756044

[13] B. Wang , D. Svetlov , D. Bartikofsky , C. E. Wobus , I. Artsimovitch , Molecules 2022, 27, 3815. 35744940 10.3390/molecules27123815 PMC9228142

[14] M. Sencanski , V. Perovic , J. Milicevic , T. Todorovic , R. Prodanovic , V. Veljkovic , S. Paessler , S. Glisic , ChemistryOpen 2022, 11, e202100248. 35103413 10.1002/open.202100248 PMC8805381

[15] Z. Fu , B. Huang , J. Tang , S. Liu , M. Liu , Y. Ye , Z. Liu , Y. Xiong , W. Zhu , D. Cao , J. Li , X. Niu , H. Zhou , Y. J. Zhao , G. Zhang , H. Huang , Nat. Commun. 2021, 12, 488. 33473130 10.1038/s41467-020-20718-8 PMC7817691

[16] C. S. Brian Chia , S. Pheng Lim , ChemMedChem 2023, 18, e202300216. 37248169 10.1002/cmdc.202300216

[17] V. Veljkovic , N. Veljkovic , J. A. Este , A. Huther , U. Dietrich , Curr. Med. Chem. 2007, 14, 441. 17305545 10.2174/092986707779941014

[18] N. Veljkovic , S. Glisic , V. Perovic , V. Veljkovic , Expert Opin. Drug Discovery 2011, 6, 1263. 10.1517/17460441.2012.638280 22647065

[19] M. Sencanski , N. Sumonja , V. Perovic , S. Glisic , N. Veljkovic , V. Veljkovic , arXiv 2020, DOI: 10.48550/arXiv.1907.02713.

[20] M. Sencanski , V. Perovic , S. B. Pajovic , M. Adzic , S. Paessler , S. Glisic , Molecules 2020, 25, 3830. 32842509 10.3390/molecules25173830 PMC7503980

[21] V. Veljkovic , J. Vergara-Alert , J. Segalés , S. Paessler , F1000Research 2021, 9, DOI 10.12688/f1000research.22149.4. PMC7202090 32419926

[22] Md. S. A. Parvez , Md. A. Karim , M. Hasan , J. Jaman , Z. Karim , T. Tahsin , Md. N. Hasan , M. J. Hosen , Int. J. Biol. Macromol. 2020, 163, 1787. 32950529 10.1016/j.ijbiomac.2020.09.098 PMC7495146

[23] I. A. Guedes , L. S. C. Costa , K. B. dos Santos , A. L. M. Karl , G. K. Rocha , I. M. Teixeira , M. M. Galheigo , V. Medeiros , E. Krempser , F. L. Custódio , H. J. C. Barbosa , M. F. Nicolás , L. E. Dardenne , Sci. Rep. 2021, 11, 5543. 33692377 10.1038/s41598-021-84700-0 PMC7946942

[24] S. Gangadharan , J. M. Ambrose , A. Rajajagadeesan , M. Kullappan , S. Patil , S. H. Gandhamaneni , V. P. Veeraraghavan , A. K. Nakkella , A. Agarwal , S. Jayaraman , K. M. Surapaneni , J. Infect. Public Health 2022, 15, 1180. 36240528 10.1016/j.jiph.2022.09.007 PMC9514006

[25] A. A. Venugopal , S. Johnson , Clin. Infect. Dis. 2012, 54, 568. 22156854 10.1093/cid/cir830

[26] J. Yuan , J. Yu , Y. Huang , Z. He , J. Luo , Y. Wu , Y. Zheng , J. Wu , X. Zhu , H. Wang , M. Li , BMC Med. 2020, 18, 204. 32731873 10.1186/s12916-020-01663-1 PMC7392643

[27] J. J. P. Stewart , Stewart Computational Chemistry, Colorado Springs, CO, 2016.

[28] J. J. P. Stewart , J. Mol. Model. 2013, 19, 1–32. 23187683 10.1007/s00894-012-1667-x PMC3536963

[29] O. Trott , A. J. Olson , J. Comput. Chem. 2010, 31, 455–461. 19499576 10.1002/jcc.21334 PMC3041641

