# Supplementary material for: SARS‐CoV‐2 PLpro Inhibition: Evaluating in Silico Repurposed Fidaxomicin's Antiviral Activity Through In Vitro Assessment
Source: ChemistryOpen. 2024 Aug 5;13(11):e202400091. doi: 10.1002/open.202400091 (PMC11564859; doi:10.1002/open.202400091)
Supplement: Supplementary file 1 — Supporting Information [file OPEN-13-e202400091-s001.pdf]

ChemistryOpen

Supporting Information

SARS-CoV-2 PL^{pro} Inhibition: Evaluating in Silico Repurposed Fidaxomicin's Antiviral Activity Through In Vitro Assessment

Sara Protić, Milica Crnoglavac Popović, Nevena Kaličanin, Olivera Prodanović, Milan Senćanski, Jelena Milićević, Kristina Stevanović, Vladimir Perović, Slobodan Paessler, Radivoje Prodanović, and Sanja Glišić*

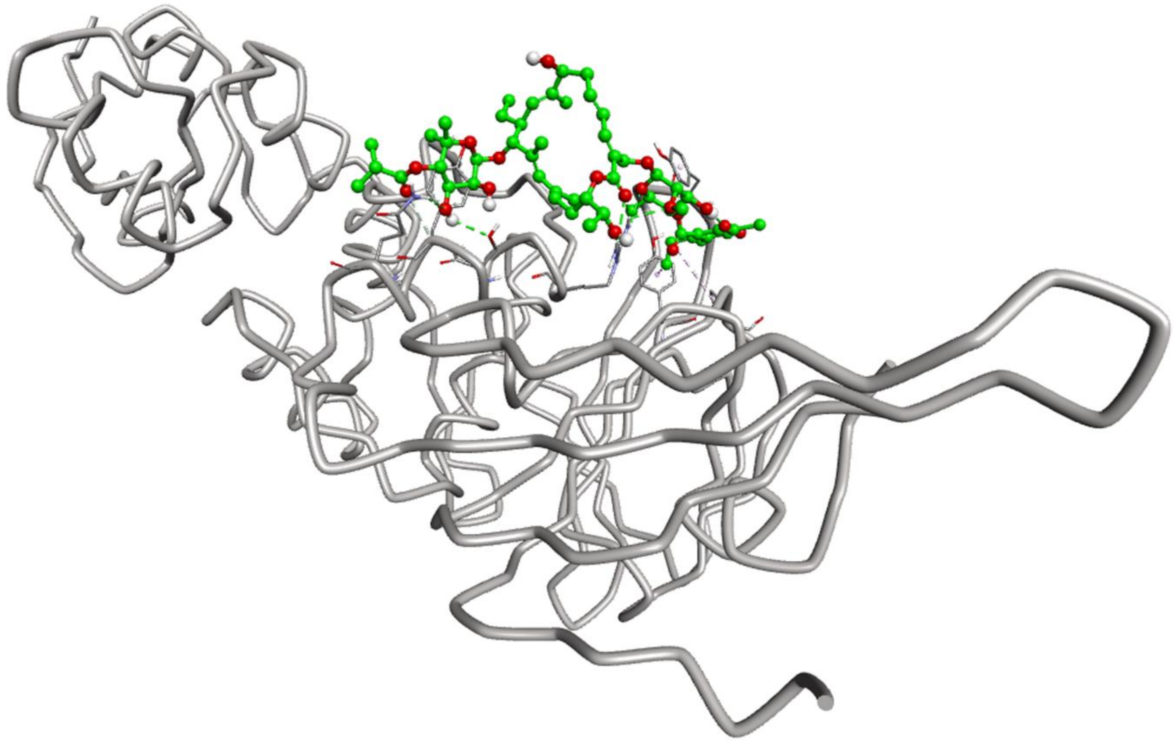

Figure S1. The whole protein with the binding site
